# Editorial for the Special Issue “Molecular Mechanism of Leukemia”

**DOI:** 10.3390/ijms241813936

**Published:** 2023-09-11

**Authors:** Jungeun An, Myunggon Ko

**Affiliations:** 1 Department of Life Sciences, Jeonbuk National University, Jeonju 54896, Republic of Korea; jan@jbnu.ac.kr; 2 Department of Biological Sciences, Ulsan National Institute of Science and Technology, Ulsan 44919, Republic of Korea; 3 Center for Genomic Integrity, Institute for Basic Science, Ulsan 44919, Republic of Korea

Hematopoiesis is the intricate process responsible for all blood cell formation and maintenance, and is tightly regulated by a myriad of intrinsic and extrinsic factors [1]. This orchestration guarantees perpetual hematopoietic homeostasis throughout an individual’s lifetime. The pivotal actors in this remarkable symphony are hematopoietic stem cells (HSCs), which reside within the bone marrow (BM). These extraordinary cells possess the unique capacity of differentiation along precisely controlled pathways, culminating in a diverse array of blood cell types. Equally vital is their self-renewal capacity, ensuring the perpetual presence of a functional HSC reservoir within the BM microenvironment.

Nonetheless, the fragile balance of normal hematopoiesis often succumbs to a spectrum of genetic and epigenetic aberrations. These deviations may disrupt the self-renewal, differentiation, proliferation, or survival of HSCs, ultimately leading to the malignant transformation of specific hematopoietic cell populations. Moreover, disruptions within the bone marrow niche that supports the requisite number and functionality of HSCs can further facilitate oncogenesis [2]. Consequently, an accumulation of aberrant, leukemic cells ensues within both the BM and peripheral blood, accompanied by a substantial suppression of normal hematopoiesis.

At the core of comprehending leukemia pathogenesis lies the precise unraveling of the roles played by genetic and epigenetic regulatory mechanisms [3]. Genetic alterations have the potential to drive neoplasia through oncogene activation or tumor suppressor gene inactivation, while epigenetic modifications can orchestrate changes in gene expression patterns without affecting the genome sequences. The intricate interplay of these regulatory processes is paramount in shaping both normal and malignant hematopoiesis. In addition to genetic and epigenetic factors, other facets also contribute significantly to leukemia development, including alterations in cellular metabolism and dysregulated interactions between hematopoietic cells and non-hematopoietic components, further fueling oncogenesis.

In this Special Issue, we proudly present a compilation of one original research article and seven reviews that delve into the diverse facets of the molecular mechanisms driving hematologic malignancies, including leukemia. In a research article by Pagliuca et al. [4], the expression of the V-domain Ig suppressor of T-cell activation (VISTA), a negative immune regulator, is found to be elevated in acute myeloid leukemia (AML) cell lines and patient samples displaying myelomonocytic differentiation, closely correlating with *NPM1* mutations. High VISTA levels at diagnosis serve as a predictor of disease recurrence in specific AML subgroups. The potential of VISTA blockade as an immunotherapeutic strategy against AML with the aim of boosting antitumor immune responses and modulating the inflammatory microenvironment is discussed. Furthermore, our contributing authors provide comprehensive reviews concerning the impact of mutations or dysregulation of key proteins such as TET proteins [5], IKAROS [6], S100A8/9 [7], NPM1 [8], and RasGRP [9] on hematologic malignancies, along with potential therapeutic strategies targeting these molecules for diagnosis and treatment. Another review addresses metabolic reprogramming in lymphomas, exploring potential signaling pathways driving these alterations and discussing therapeutic strategies targeting metabolic pathways [10]. Additionally, insights into the role of the Hedgehog signaling pathway during normal T-cell development and in T-cell acute lymphoblastic leukemia (T-ALL) pathogenesis are reviewed, along with potential therapies targeting the pathway in T-ALL [11].

Comprehending the fundamental molecular basis that governs both normal and malignant hematopoiesis is essential for more rational and effective therapeutic interventions. While a substantial amount of work remains, this compilation of cutting-edge research will contribute to unraveling the complexities of the diseases, improving patient outcomes, and advancing fundamental knowledge, ultimately granting safer and more effective personalized treatments for this neoplasm. Finally, the Guest Editor extends their deep appreciation to the dedicated authors whose invaluable contributions have enriched this endeavor, as well as to the Editorial Board for their unwavering support.
